# Examining the Relationship Between Hair Cortisol With Stress-Related and Transdiagnostic Subclinical Measures

**DOI:** 10.3389/fpsyt.2021.746155

**Published:** 2021-11-11

**Authors:** Pilar Torrecilla, Neus Barrantes-Vidal

**Affiliations:** ^1^Departament de Psicologia Clínica i de la Salut, Facultat de Psicologia, Edifici B, Universitat Autònoma de Barcelona, Barcelona, Spain; ^2^Departament de Salut Mental, Sant Pere Claver—Fundació Sanitària, Barcelona, Spain; ^3^Centre for Biomedical Research in Mental Health (CIBERSAM), Madrid, Spain

**Keywords:** hair cortisol, stress, hypothalamic-pituitary-adrenal axis, transdiagnostic, subclinical symptoms, schizotypy

## Abstract

**Background:** Hair cortisol concentrations (HCC) provide a retrospective examination of long-term cortisol production as a measure of the hypothalamic-pituitary-adrenal (HPA) axis functioning, one of the major neural systems implicated in mediating the effects of stress on mental illness. However, evidence about the relationship between HCC with stressors and symptoms is scattered. In the present study, we aimed to examine the association between HCC and a wide range of stress-related and transdiagnostic subclinical measures in a sample of non-clinical young adults with a wide distribution of schizotypy.

**Methods:** A total sample of 132 non-clinical young adults recruited at college and technical schools oversampled for schizotypy scores were assessed on distal and proximal stressful experiences, appraisals of stress, traits and symptoms of the affective, psychosis and dissociation spectrums, as well as stress-buffering measures, and provided 3 cm-hair samples.

**Results:** No significant associations were found between HCC and any of the stress-related and subclinical measures. Only suspiciousness and disorganization showed a trend for a positive association with HCC but the magnitude was small.

**Conclusions:** The present findings support previous studies indicating an overall lack of concordance between a broad range of stress-related and (sub)clinical phenotypic measures with hair cortisol. This study examined for the first time the relationship of HCC with the non-clinical expression of the psychosis spectrum, that is, schizotypy, which complements previous studies on clinical high risk and established psychosis and offers a promising strategy for studying possible HPA dysfunctions characterizing the subclinical psychosis continuum without the confounds associated to clinical psychosis.

## Introduction

The Hypothalamic-pituitary-adrenal (HPA) axis is one of the major neural systems implicated in mediating the effects of stress on mental illness. This axis acts in response to stressors by releasing glucocorticoid cortisol, thereby affecting brain function and facilitating physiological and behavioral responses to threats. The normal functioning of the HPA axis involves not only its activation under high stressful situations, but also the activation of a negative feedback-loop that stops cortisol secretion in the absence of stressors (1). However, both the activation and inactivation of the HPA axis can be susceptible of dysregulation, which has been widely connected with the development of mental disorders (2).

Cortisol concentrations have been widely analyzed from blood serum, saliva or urine. However, these measurements have only been capable of reflecting acute or short-term responses to stress, with remarkable intra- and inter-individual variability and day fluctuations. In contrast, hair cortisol, which enables a retrospective examination of long-term cortisol production, has recently been proposed as a more accurate measure of chronic stress (3). As hair grows ~1 cm per month (4), a 3 cm-sample of hair has been considered a reliable and usable segment to reflect the cortisol production of the last 3 months from the strand collection. Moreover, it is a non-invasive technique that avoids any further stress associated to the sampling procedure and can be easily stored and transported. Although hair cortisol has been found to be subject to developmental and seasonal variations (5), a considerable degree of intraindividual stability has been assumed and validated so far (6), thus constituting a promising method for the retrospective and stable assessment of cortisol functioning.

Research has provided emerging evidence on hair cortisol concentrations (HCC) alterations in clinical populations, mostly in affective and anxiety disorders and, to a lesser extent, in psychosis. However, the direction of these alterations is yet to be elucidated and there is some inconsistency across studies (7). Depression has been associated with HPA axis hyperactivity (8) and elevated short-term cortisol response (9). Nonetheless, when examining the long-term activity of the HPA axis assessing HCC in depressed patients, results are unclear. A recent meta-analysis by Psarraki and colleagues (10) shows that most studies found no significant differences in HCC between Major Depression Disorder (MDD) patients and controls (11–13), although one study (14) showed higher HCC and two studies (15, 16) found lower HCC in MDD patients. Nonetheless, higher levels of HCC were found in first episode compared to recurrent depression (17, 18) or when there is comorbidity between MDD and an anxiety disorder (19). Bipolar Disorder patients seem to present higher HCC compared to controls (20–22) as reported in the meta-analysis by Koumantarou Malisiova and colleagues (7), and again, HCC were higher for those with psychiatric comorbidities (23). In contrast, decreased HCC have been found in patients with Generalized Anxiety Disorder (GAD) (24) or other anxiety disorders such as Post Traumatic Stress Disorder (PTSD) (25), although other studies have failed to find an association (16, 19). Results regarding stress-related symptoms in non-clinical populations are scant and have yielded mixed findings. HCC in adolescents (26, 27) or adult workers (28) have been positively associated with depressive symptoms, but lower HCC (29) or no association (30, 31) have also been reported. Studies examining the relationship between hair cortisol and anxiety in non-clinical participants did not find any significant association (29, 31).

Consistent with the current focus on a broader transdiagnostic approach to etiological research in psychopathology [e.g., (32, 33)], studies on phenotypes that have been traditionally less associated to stress-sensitivity, such as the psychosis spectrum, are starting to emerge. There is robust evidence that psychotic-spectrum disorders are associated to childhood adversity (34–37), heightened stress-sensitivity (38–40), and elevated HPA activity (1, 41), suggesting the HPA axis as a relevant mediator of the effects of stress on psychotic symptoms (42, 43). Specifically, elevated HCC has been found in clinical samples of schizophrenia (20, 21), First Episode of Psychosis (FEP) individuals (44) and populations at clinical risk for psychosis (45). However, no studies have examined yet the association of HCC with the non-clinical manifestations of the extended psychosis phenotype, that is, schizotypy traits. Schizotypy is a multidimensional construct that represents the underlying liability for psychosis-spectrum psychopathology expressed across a broad range of personality, subclinical and clinical psychotic features (46).

Exposure to early life adversity has been associated to HPA axis dysregulation, which, in turn, has been found to be robustly associated to elevated risk for developing mental illness. Thus, most current etiological models support that prolonged and/or severe stress exposure in highly sensitive developmental periods (i.e., childhood) disrupts psychobiological stress regulation mechanisms resulting in a process of behavioral and biological sensitization by which the individual manifests an enhanced stress sensitivity to subsequent minor adversities in adulthood (47, 48). Early adversity has been associated to HCC in clinical populations with a psychotic, affective, personality and/or anxiety disorder (20, 49), in individuals clinically at-risk for psychosis (45) and also in non-clinical populations (50–53). However, the last meta-analysis from Khoury and colleagues (54) assessing the strength and direction of the relationship between adverse experiences and HCC (including clinical and non-clinical samples) found two classes of studies: a first one including a majority of studies (*N* = 24) showing a positive association between adversity and HCC, and a second minor group of studies (*N* = 4) showing lower levels of hair cortisol in those exposed to adverse experiences. All studies in the latter group were assessing childhood maltreatment and showed a moderate effect size; in contrast, the first class of studies included a variety of adversities other than childhood maltreatment (e.g., exposure to natural disasters or domestic violence) and showed a small effect size. This pattern of mixed results might be consistent with theories positing both types of HPA axis alteration following adversity; hyper-activity in the short-term and hypo-activity in the long-term (55). It also indicates the need of differentiating among types of adversities and populations when studying the effects of adversity on the HPA axis.

Another critical question is the association between proximal psychosocial stressors and HPA axis function. A major issue is that the relationship between subjective (i.e., perceived stress, subjective impact of life events) and objective (i.e., cortisol, number of life events) measures of stress has not been coherent across studies and yielded mixed findings. The meta-analysis by Stalder and colleagues (25) indicated no association between subjective perceived stress and HCC across populations exposed to different levels of chronic stress. More recent studies have also failed to find significant associations (30, 56–59), except for Ling and colleagues (60), who found a negative relationship between perceived stress and HCC in African non-clinical mothers (with a quite reduced sample size), and Xu and colleagues (29), who reported, in contrast, a positive association in a Chinese sample of healthy adolescents differing in stress exposure (incarcerated vs. attending regular high school). On the other hand, another widely used measure to assess “objective” stress is the quantification of recent life events. This has been mostly studied in adolescent samples, and mixed results have been found. Shapero and colleagues (26) did not find any association between HCC and number of life events, whereas Xu's (27) and Karlén's (61) studies reported a positive association and Sierau's (31) a negative one. Finally, Cullen and colleagues (62) reviewed the literature examining the concordance between naturally-occurring psychosocial stressors, both distal (trauma) and proximal (perceived stress and life events), with cortisol across clinical and at-risk psychosis populations. Although all types of cortisol measurements were included in the meta-analysis, a poor correlation [*r* = 0.05 (95% CI: −0.00 to 0.10), *p* = 0.059] was found between stressors and cortisol measures (including HCC).

Research so far has only studied the relationship between HCC and the occurrence of stressful life events, but any of those studies has reached to study the whole spectrum of life events, comprising both adverse as well as positive ones (e.g., getting married). Additionally, there is no research on the association between the HCC and the degree of subjective impact (positive or negative) of these life events on the individual. Given large individual differences in stress-related genetic make-up (e.g., BDNF, FKBP5, COMT, 5-HTTLPR), temperamental traits (e.g., neuroticism, harm avoidance), gene-environment interactions, and idiographic contextual factors, it is expected that the same amount of life events can impact very differently on individual's appraisals [e.g., (63)], and, therefore, on levels of HPA axis dysregulation.

The association between neuroticism, a temperamental trait defined by heightened stress reactivity (64), and HCC has only been examined in a twin study, showing no significant correlation (30). Similarly, little is known about the association of HCC with protective factors related to coping with stress. The buffering hypothesis poses that the presence of social support might help buffering the potential deleterious effects of stressful situations (65). However, the biological impact of the effects of social support on stress and, thus, on the HPA axis, has been scarcely investigated. Stalder and colleagues (25) did not find any significant association between HCC and social support in their meta-analysis, whereas Iob and colleagues (66) reported that low social support was associated to high levels of hair cortisol. In contrast, a recent study from Yang and colleagues (67) found a positive association between HCC and the amount of social support in a sample of schizophrenia patients; however, social support moderated the relationship between stressful life events and HCC by attenuating the effects of life events on cortisol response, thus providing biological support to the buffering hypothesis.

In summary, there is a major concern about the lack of “psychoendocrine covariance” between subjective and objective measures of stress. As referred in Stalder's meta-analysis (25), no consistent associations between HCC and self-reports of perceived stress, social support and depression symptoms emerged in the most recent literature. Moreover, this literature is very scattered in terms of the types of constructs, measures and samples used. Importantly, most studies have examined a very narrow range of stress-related and/or phenotypic variables presumed to be associated to HCC within the same sample—in fact, only meta-analytic studies have offered an integrated perspective of the association of stress and psychopathology measures with HCC. Therefore, the present study aimed to investigate the association between HCC with a broad range of psychosocial stressors (childhood adversity, recent life events) and stress-related measures (perceived stress). Importantly, the stress-related measures used covered both the “objective” report of threatening and stressful life events as well as the “subjective” appraisal of stress impact. In addition, and consistent with current evidence on the relevance of heightened stress-sensitivity as a relevant transdiagnostic mechanism, the association of traits and symptoms of the affective (neuroticism, depression, anxiety), psychosis (suspiciousness, schizotypy) and dissociation spectrums with HCC was examined. Finally, stress-buffering factors (social support) were also examined in a non-clinical sample of young adults oversampled for high levels of schizotypy (i.e., the behavioral liability to psychosis). Examining non-clinical participants with a wide distribution of behavioral risk for psychosis ensures including a representation of individuals with a phenotype that has been consistently associated with high stress exposures (36, 37) and heightened stress-sensitivity (1, 38, 39, 68), which should enrich the variability in the constructs of interest (stress exposures, HCC and stress-related phenotypic features). Consistent with evidence supporting the dimensional conceptualization of psychopathology and the transdiagnostic relevance of the HPA-axis dysregulation, we expected a general pattern of associations between HCC with the stress-related measures and the different subclinical spectra in a non-clinical sample.

## Methods

### Participants

The sample of this study consisted of 132 non-clinical young adults (mean age = 27.86, SD = 3.07, range = 26.07) belonging to the ongoing Barcelona Longitudinal Investigation of Schizotypy Study (BLISS) (68–70).

At T1, a large pool of 547 unselected college students and 261 technical school students were initially screened with self-report questionnaires (68, 69). A subsample oversampled with elevated scores on both positive and negative schizotypy factors (to ensure enough variance in the measures of interest) from the Wisconsin Schizotypy Scales (WSS-S) (71, 72), the Schizotypal Personality Questionnaire (SPQ) (73) and the Community Assessment of Psychotic Experiences (CAPE) (74) was selected to conduct in-depth examinations comprising a wide range of interview, questionnaire, and experience sampling methodology measurements. At T2, 214 college and 39 technical school students were reassessed (1.7 and 0.4 years later, respectively). At T3, due to funding constraints, we invited to participate a reduced subsample that retained the original distribution of scores; 103 college and 31 technical school participants were reassessed (1.4 and 1.7 years later, respectively). Of these, 89 college participants were re-assessed at T4 (1.3 years later). Finally, at T5 we were able to successfully reassess 168 (79%) of the college students assessed at T2 7.8 years later (and 3.2 years after T4). In addition, we reassessed 26 (77%) of the technical school participants at T3 2.4 years later. Therefore, a total of 194 participants were assessed at T5, the study phase when hair samples were collected.

At T5, 132 out of the 194 participants (112 were college students and 20 technical school students) were included in the present cross-sectional study. Participants completed self-report questionnaires (except for childhood adversity, which was available from T1) and provided 3 cm-hair samples. Sample size varies for some measures given that three of the participants who provided hair samples did not complete the questionnaire assessment (*N* = 129). All participants provided written informed consent to participate.

### Materials and Procedure

#### Stress-Related Measures

The Perceived Stress Scale (PSS) (75) enquires about the level of stress perceived by participants during the last month. It is a self-reported questionnaire of 14 items that provides a total score of perceived stress (Cronbach alpha = 0.86).

Two complementary measures of life events were used. The List of Threatening Events (LTE) (76) consists of 20 items (YES/NO) asking about adverse life events that might have occurred during the last year. In contrast, the Life Events Survey (LES) (77) includes 57 life events comprising a full range of experiences from negative to positive plus three blank spaces for other events. Forty-seven of them refer to general life events and 10 of them are academic-related. We removed one academic-related item (“Academic probation”) given that there is not an equivalent of it in the Spanish education system. Participants rate both the occurrence (YES/NO) of the event and the impact it caused on them, capturing both a negative as well as a positive valence by using a Likert scale ranging from −3 (very negative) to +3 (very positive). In the present study, only the subjective mean impact of life events was included (Cronbach alpha = 0.78).

The Childhood Trauma Questionnaire Brief (CTQ-B) (78) data was available for participants from the baseline data (T1) collection of the BLISS study. It is a self-reported measure covering 28 items rating the severity of emotional abuse and neglect, physical abuse and neglect and sexual abuse. A total score of childhood trauma comprising all the subscales is used in the present study (Cronbach alpha = 0.85).

#### Affective Symptoms and Personality

The Beck Depression Inventory-II (BDI-II) (79) was used to assess depressive symptoms. It has 21 items including a range of affective, behavioral, cognitive and somatic symptoms (Cronbach alpha = 0.88). Anxiety was measured with the Anxiety scale of the Symptom Checklist-90-Revised (SCL-90-R) (80). This scale consists of 10 items that can be answered with a Likert-scale from 0 to 4 (Cronbach alpha = 0.82).

Neuroticism was measured using the 8-item neuroticism subscale of the Big Five Inventory (BFI) (81), a 44-item inventory that measures five predominant dimensions of personality (extraversion, convenience, consciousness, neuroticism and openness) through 5-point Likert scales ranged from “strongly disagree” to “strongly agree” (Cronbach alpha = 0.83).

#### Psychosis and Dissociation Spectrum Personality Traits and Experiences

In terms of paranoid personality, the Suspiciousness Scale of the Schizotypal Personality Questionnaire (SPQ) (73) was used (Cronbach alpha = 0.70). Schizotypy personality traits were assessed with the short forms of the Wisconsin Schizotypy Scales (WSS-S) (71, 72), from which participants were assigned positive and negative schizotypy factor scores (68, 69) and the Multidimensional Schizotypy Scales Brief (MSS-B) (82), a 39-item measure which provides positive, negative and disorganized scores of schizotypy. The MSS-B was introduced in the protocol assessment once the sampling had already started, so only 59 (40 college and 19 technical school students) participants have data for the MSS-B. Of note, the MSS-B has demonstrated to overcome limitations associated with existing measures of schizotypy such as unclear conceptual framework, outdated items, ethnical/sex differences, or exclusion of disorganized schizotypy and to show good internal reliability and construct validity (83, 84). Data of the MSS-B for the disorganized dimension (Cronbach alpha = 0.75) and the factorially-derived dimensional scores based on the WSS for the positive and negative dimensions were used.

Dissociation was assessed using the Dissociative Experiences Scale (DES–II) (85), a 28-item instrument where participants are prompted to answer from a range of 0% (never) to 100% (all the time) the frequency of each of the dissociative experiences presented (Cronbach alpha = 0.82).

#### Stress-Buffering Factors

The Multidimensional Scale of Perceived Social Support (MSPSS) (86) is a 12-item scale designed to measure perceived social support from three sources: Family, Friends, and a Significant Other. The total sum of perceived social support has been used in this study (Cronbach alpha = 0.92).

#### Hair Cortisol

Hair strands were cut as close as possible from the posterior vertex area of the head. Specifically, cortisol concentrations were determined from the 3 cm hair segment most proximal to the scalp. As hair grows in average 1 cm per month (4), the samples represented the cortisol mean levels from the last 3 months. All samples were stored in aluminum foil at room temperature until the extraction procedure. Then, the samples were washed twice for 1 min with 10 ml of isopropanol and completely air dried at room temperature. After that, samples were further cut into fragments of ~2−3 mm and mixed with 1.6 methanol overnight with continuous rotation. All hair samples weighted within the recommended range required for immunoassay analyses [i.e. 5–50 mg; (61, 87)]. Methanol was recovered in a new clean glass tube. The extraction was repeated, and the recovered methanol was pooled and dried up under a nitrogen stream. Extracted cortisol was re-suspended in 200 μl of PBS and assayed for cortisol. HCC were then determined by a radioimmunoassay procedure. Also, participants were prompted to answer whether they have ever dyed their hair and how many times do they wash their hair in a week.

#### Statistical Analyses

Statistical analyses were performed using the Statistical Package for the Social Sciences (SPSS), Version 22.0 software (88). ANOVA was used to test differences in hair cortisol concentrations for academic group, sex, age and hair dye, and Pearson correlations were used to test associations between hair cortisol and hair wash frequency as well as the different environmental, clinical and psychosocial measures.

## Results

One out of 132 participants was excluded because of abnormally increased HCC (244.6 pg/mg) compared to the rest of the sample. As shown in Table 1, mean cortisol levels of the final sample of 131 participants were 6.22 pg/mg (s.d. 4.93), ranging from 1.30 to 39.70 pg/mg. Males (*N* = 22) showed mean cortisol levels of 8.20 pg/mg (s.d. 8.31) and females (*N* = 109) 5.81 pg/mg (s.d. 3.86), with no significant differences (*p* = 0.20). No differences between college students and technical school students were found on HCC (*p* = 0.22). Therefore, the two samples were combined to perform the correlational analyses of HCC with the measures of interest. HCC were not affected by hair dye (*p* = 0.542) or frequency of washing (*p* = 0.146).

**Table 1:** Descriptive data of the sample and hair cortisol concentrations (HCC).

		**HCC differences**	
		**Statistic test**	***p*-value**
HCC (M, SD)	6.22 pg/mg, 4.93	–	–
**Demographics**			
Age (M, SD)	27.84, 3.08	–	–
Sex (N)		^a^*t* = 2.09	0.038^*^
Male	22		
Female	109		
**Hair-related variables**			
Hair dye (*N*)		*t* = 0.612	0.542
Yes	22		
No	109		
Washing frequency (M, SD)	3.05, 1.76	^b^*r =* 0.128	0.146

a *T-Test*,

b *Pearson Correlations*.

* *p < 0.05. M = Mean, SD = Standard Deviation*.

Correlational analyses (Table 2) showed no significant associations between HCC and any of the psychosocial or phenotypic measures. Only suspiciousness and disorganization showed a trend for an association with HCC with a positive correlation of *r* = 0.149 (*p* < 0.10) and *r* = 0.220 (*p* < 0.10), respectively.

**Table 2:** Correlational analyses between stress-related and subclinical measures with HCC.

	**M, SD**	***r***	***p-value***
**Psychosocial stress**			
**Distal**			
Childhood Trauma	33.91, 8.03	0.094	0.287
**Proximal**			
Perceived stress scale	20.19, 7.63	−0.006	0.949
Life events			
List of threatening events (LTE)	2.28, 1.97	0.04	0.656
Subjective impact of life events (LES)	0.51, 1.04	−0.026	0.772
**Affective, psychosis and dissociation spectrum traits and symptoms**			
Neuroticism	2.69, 0.61	−0.097	0.276
Depression	5.70, 6.66	−0.046	0.607
Anxiety	5.41, 4.75	0.004	0.964
Suspiciousness	1.57, 1.67	0.149	0.094^+^
Positive schizotypy	0.93, 1.5	0.13	0.326
Negative schizotypy	2.13, 2.04	−0.027	0.839
Disorganized schizotypy	0.78, 1.45	0.22	0.094^+^
Dissociation	7.89, 7.19	0.043	0.63
**Stress-buffering measures**			
Social support	71.97, 13	−0.01	0.909

+ *p < 0.10. M = Mean, SD = Standard Deviation. Data was available for N = 131 participants for the Childhood Trauma Questionnaire (CTQ) assessed at T1, whereas three participants that provided hair samples at T5 did not complete the questionnaire assessment at T5 (N = 129)*.

## Discussion

To the best of our knowledge, this is the first study examining a wide range of stress-related variables including both risk and protective factors, subjective and objective measurements, and employing a transdiagnostic perspective of stress-related phenotypes in a sample of non-clinical young adults. Moreover, this is the first study examining the relationship of HCC with the non-clinical expression of the psychosis spectrum, that is, schizotypy and paranoid personality traits along with other stress-related phenotypes.

Results showed no significant associations between HCC and the different psychosocial stress measures. Contrary to the hypothesis, but consistent with previous studies, depression, anxiety and neuroticism were not associated with HCC. In contrast, suspiciousness and disorganization, both strongly related to affective symptoms, showed a trend toward a significant association with HCC, although the magnitude was small in both cases. Suspiciousness consistently shows a large association with depression, low self-esteem, and neuroticism (89–91). Similarly, the disorganized dimension of schizotypy shows the strongest association with negative affect, depression and anxiety compared to positive and negative schizotypy (92, 93). Interestingly, the disorganization dimension measured by the MSS scale does not include items that explicitly assess affect, but disrupted affect (e.g., excessive negative affect and inappropriate affect) is possibly equally relevant to this dimension as disruptions of thought, speech and behavior (93). It is likely that the fact that participants were oversampled for schizotypy to ensure sufficient variability in these traits allowed to capture this trend for an association—something that might be missed given the skewed nature of these traits in non-clinical samples. Finally, from a transdiagnostic psychopathology perspective, it is attractive to speculate that weak trends only emerged for the most severe traits of mental the disorder spectrum. Both suspiciousness and disorganization traits entail an intense and enduring disruption of a broad range of cognitive, affective and behavioral aspects, usually associated to mental suffering and impairment. Nonetheless, the weak associations of these constructs with hair cortisol calls for further examination of the association of HCC with a broad transdiagnostic range of psychopathology dimensions across non-clinical schizotypy and clinical high-risk populations.

Overall, the present findings add to previous studies showing a lack of psychoendocrine covariance between the long-term hair cortisol measurement with psychosocial stressors, symptomatology and other stress-related phenotypes in non-clinical samples. It has been suggested that this unexpected finding might be related to the fact that samples from studies assessing stress and cortisol usually comprise individuals that have been exposed to either very high (clinical) or very low (“super normal” controls) levels of stress (25). However, the present study aimed to avoid this limitation by employing a non-clinical sample encompassing a wide range of variance along schizotypy dimensions in order to ensure the presence of sufficient variability in the constructs of interest (i.e., stress-related measures, traits and subclinical symptoms). Another hypothesis that has been raised to account for this poor concordance is that a significant hair cortisol elevation might require an intense and persistent exposure to stress (25). Cross-sectional studies might not be the most adequate to observe persistent or repeated exposure to stress. In that sense, further research based on longitudinal assessments is needed to examine whether a notable HPA axis dysfunction is observable after persistent exposure to stressful situations. Also, inconclusive findings between HCC and stress-related measures have been attributed to some methodological aspects. For instance, the use of different analytic methods to obtain HCC. Whilst most studies have been using traditional immunoassay methods and exhibiting great sensitivity, liquid chromatography-mass spectrometry (LCMS) based-assays are also employed, which might be yielding heterogeneous results in meta-analytic work including both techniques (7, 94). Another methodological limitation has been attributed to the discrepancy between timeframes captured by hair cortisol and self-reported stress-related measures (7, 95). However, there is no consensus as to whether this is actually a limitation, as hair cortisol is considered to be a “long-term,” “stable” or even a “chronic” biological indicator of stress. Compared to previous “short-term” techniques such as saliva, blood serum or urine, HCC represent a long-term measurement of cortisol levels as 3-cm hair samples provide mean cortisol levels from the last 3 months. Importantly, intraindividual stability has been demonstrated in most studies assessing the correlation between repeated hair cortisol assessments (usually two to three time points) across periods of one (96), two (97), three (98, 99), four (97), six (5, 6) and twelve (97) months in adult populations. Nonetheless, all those studies employed non-clinical populations, usually healthy adults or unselected college students, and only two of them (98, 99) focused on individuals explicitly exposed to certain levels of stress (e.g., postpartum period or high-risk community). Findings in depression on higher HCC in the first but not in the recurrent episodes (17, 18) and the inconsistent association of HCC and childhood trauma (54, 55), raise concerns about the validity of hair cortisol as a measure of chronic stress levels.

Of note, a major concern has been the degree of association between subjective and objective psychosocial stressors with biological markers of stress such as HCC. It has been suggested that exposure to early adversities in life predisposes individuals to overreact to subsequent stressful experiences in adulthood due to a biological and behavioral stress sensitization process that, in turn, contributes to the risk of mental illness in general, and psychosis in particular (39, 40, 100, 101). Moreover, the neural diathesis stress model poses that the HPA axis plays a major role as a mediator of the effects of stress on the development of, for instance, depression (102) and psychosis (1, 103). As hair cortisol has been considered a reliable biomarker to capture the HPA function, it is expected to find disrupted HCC in those individuals that have experienced higher levels of psychosocial stress. Nonetheless, only scarce research has found an association between early adversity, life events or perceived stress and hair cortisol, and the direction of the associations has been contradictory among studies. Cullen and colleagues (62) reviewed studies examining the concordance between psychosocial stressors and cortisol measures in healthy controls, individuals at clinical high-risk for psychosis and with clinical psychosis, and only weak correlations were found. The present study complements the studies reviewed (62) by focusing on non-clinical individuals with psychometric high risk, although more research on the psychoendocrine covariance across the psychosis continuum is needed. Furthermore, the present study included a critical measure not included in previous studies on HCC: the subjective appraisal of the impact of stressful life events. It has been suggested that the effect of a life event on an individual may result more from the appraisal of the perceived impact than the life event itself (104, 105), and most likely, the biological impact of such event on the stress system depends on whether individuals do perceive that event as stressful However, no association was found with either the “objective” number of life events or the “subjective” appraisal. A possible explanation for this lack of associations might be the large individual variation in stress-sensitivity. Stress-sensitivity has been defined as a developmental phenomenon in which individuals tend to be highly reactive to both low and extremal levels of stress that emerges from relations between genetic, temperamental and contextual factors (48, 106). Thus, further gene—environment and person—environment interaction studies are needed to better understand the complexity of stress-sensitivity and to integrate individual differences in this trait in designs mostly based on group-level associations.

In conclusion, this study supports previous studies indicating an overall lack of concordance between a broad range of stress-related and (sub)clinical symptom measures with hair cortisol. This study examined HCC in individuals oversampled for schizotypy traits (i.e., psychometric high risk) for the first time, which complements studies on clinical high risk for psychosis and offers a promising strategy for studying possible HPA dysfunctions characterizing the subclinical psychosis continuum without the confounds associated to clinical status and medication. Further studies examining hair cortisol and its relationship with stress-related symptoms, phenotypes and psychosocial stressors in non–clinical samples exposed to different levels of stress are needed. Also, longitudinal studies might help to study stress persistence as a possible essential feature for HPA axis disruption. As posed by the RDoC framework (32), an integrated and translational approach from an endocrine, genetic and psychological level, as well as the study of their interaction with environmental influences, is needed to completely understand the mechanisms underlying HPA axis dysfunction and its relationship with mental health.

## Data Availability Statement

The raw data supporting the conclusions of this article will be made available by the authors, without undue reservation.

## Ethics Statement

The studies involving human participants were reviewed and approved by Ethics Committee of the Universitat Autònoma de Barcelona (Comissió d'Ètica en l'Experimentació Animal i Humana (CEEAH); https://www.uab.cat/web/investigacio-humana/presentacio-1345713929159.html). The patients/participants provided their written informed consent to participate in this study.

## Author Contributions

NB-V conceived the study, acquired funding, administered and supervised the project and data acquisition, contributed and revised the manuscript. PT analyzed the data and wrote the manuscript. All authors contributed to the article and approved the submitted version.

## Funding

This work was supported by the Spanish Ministry of Science, Innovation and Universities (Grant number PSI2017-87512-C2-1-R) and the Comissionat per a Universitats i Recerca of Generalitat de Catalunya (Grant No. 2017SGR1612). PT was supported by the Spanish Ministry of Science, Innovation and Universities (Grant No. PRE2018-085299).

## Conflict of Interest

The authors declare that the research was conducted in the absence of any commercial or financial relationships that could be construed as a potential conflict of interest.

## Publisher's Note

All claims expressed in this article are solely those of the authors and do not necessarily represent those of their affiliated organizations, or those of the publisher, the editors and the reviewers. Any product that may be evaluated in this article, or claim that may be made by its manufacturer, is not guaranteed or endorsed by the publisher.
